# Upregulation of FNDC5 gene expression in C2C12 cells after single and combined treatments of resveratrol and ATRA

**DOI:** 10.1186/s12944-019-1128-y

**Published:** 2019-10-22

**Authors:** Elahe Abedi-Taleb, Zahra Vahabi, Ehsan Sekhavati-Moghadam, Leila Khedmat, Shima Jazayeri, Ali Akbar Saboor-Yaraghi

**Affiliations:** 1 Department of Cellular and Molecular Nutrition, School of Nutritional Sciences and Dietetics, Tehran University of Medical Sciences, Tehran, Iran; 2 Department of Geriatric Medicine, Ziaeian Hospital, Tehran University of Medical Sciences, Tehran, Iran; 3 Memory and Behavioral Neurology Division, Roozbeh Hospital, Tehran University of Medical Sciences, Tehran, Iran; 4 Department of Cardiology, Ziaeian Hospital, Tehran University of Medical Sciences, Tehran, Iran; 5 Health Management Research Center, Baqiyatallah University of Medical Sciences, Tehran, Iran; 6 Department of Nutrition and Biochemistry, School of Public Health, Iran University of Medical Sciences, Tehran, Iran; 7 Department of Immunology, School of Public Health, Tehran University of Medical Sciences, Tehran, 141613151 Iran

**Keywords:** FNDC5, Thermogenesis, Retinoic acid, Resveratrol

## Abstract

**Background:**

Irisin is a newly discovered myokine that secreted from skeletal muscle cells. Several studies showed that irisin involves in thermogenesis and increases the expression of browning markers such as uncoupling protein-1 that in turns induces the conversion of white adipose tissue to brown fat. Resveratrol (Res) and all-trans retinoic acid (ATRA) can also upregulate the expression of thermogenesis genes. In the present study, the effects of single and combined treatments of Res and ATRA on fibronectin type III domain containing 5 (FNDC5) gene expression was explored.

**Methods:**

The mouse myoblasts, C2C12 cells, were seeded in 6-well plastic plates and cultured in DMEM media. After differentiation, in a pilot study, C2C12 myotubes were treated with different concentrations of Res and ATRA for 12 h. The best result was obtained by treatment of 1and 25 μM of Res and 1 μM of ATRA. Then the main study was continued by single and combined treatment of these compounds at chosen concentration. After treatments, total RNA was extracted from C2C12 cells. Complementary DNA (cDNA) was generated by the cDNA synthesis kit and FNDC5 mRNA expression was evaluated by the real-time PCR method.

**Results:**

The FNDC5 gene expression in C2C12 myotubes of alone-treated with 1 μM, 25 μM Res and 10 μM ATRA did not change compared to vehicle group. However, in combination-treated the expression of FNDC5 gene was significantly increased compared to vehicle group.

**Conclusion:**

This is the first evidence that Res and ATRA can regulate FNDC5 gene expression in C2C12 myotubes. More investigations are necessary to explore the therapeutic effects of these nutrients in obesity, diabetes, cardiac and neurovascular disease.

## Background

The skeletal muscle is an endocrine organ and is able to generate and release some active proteins called myokines [1]. It has been shown that irisin, a newly discovered myokine, secretes into the circulation after cleavage from the fibronectin type III domain containing-5 (FNDC5 (gene and numerous researchers highlighted the muscle intervention in metabolism [2–5].

Irisin/FNDC5 identified as a cytokine which imitates the beneficial effect of exercise on adipose tissue [6]. FNDC5 is stimulated by peroxisome proliferator-activated receptor γ coactivator-1α (PGC-1α) and expressed in adipocyte and many tissues such as kidney, liver, heart, and lung [7] but the expression of FNDC5 in muscle tissue is 200 fold greater than in adipose tissue [8]. Irisin is a potentially candidate marker for heart and neurovascular disease [9–11]. PGC-1α identified as a regulator of mitochondrial function. In addition, this component coactivates peroxisome proliferator-activated receptor (PPAR) α and PPARγ. There is an enriched expression of PGC-1α in brown adipose and other thermogenesis tissues. Cold exposure and fasting induce this master regulator in human metabolism. Activation of uncoupling protein-1 (UCP-1), is mediated by PGC-1α through FNDC5 which induces thermogenic program, the biological conversion of white adipocyte (WAT) into brite/beige adipocyte (BAT) [12, 13]. The thermogenic program is an attractive target in the treatment of obesity. BAT plays an important role in regulation of body weight through increasing the energy expenditure [14]. Obese people may have various diseases including type 2 diabetes, heart disease and cancer [15]. Obesity is a serious health problem and linked to genetic predisposition and sedentary lifestyle [16]. Although the considerable focus has been put on the understanding the etiology of obesity and diabetes, the role of several factors from skeletal muscle is unknown [17]. A recent study investigated the signal generated by peripheral tissues including fat and skeletal muscle [1]. A very recently published study showed that FNDC5 expression is induced by ATRA treatment [18]. Several studies are investigated in irisin different functions. Nevertheless, the potential role of irisin in humans is unclear and there are still many questions in the exact role of irisin in metabolism.

ATRA is an active derivative of vitamin A and Res is a type of natural phenol which can be found in some foods including the skin of grapes, blueberries, raspberries, and mulberries [19, 20]. Recent studies revealed the signaling pathway of Res and ATRA effects on PGC-1α activity [21–29]. It has been shown that ATRA and Res regulate thermogenesis by inducing the expression of UCP-1 [30–32].

The present research investigated the effects of single and combined treatments of Res and ATRA on the FNDC5/ irisin gene expression in C2C12 cells.

## Materials and methods

### Cell culture

C2C12 mouse myoblasts were purchases from a cell bank (Pasteur Institute, Tehran, Iran) and cultured in DMEM (Gibco, Invitrogen, UK) containing 1000 mg/L glucose, supplemented with 10% heat-inactivated fetal bovine serum (Gibco, Invitrogen, UK) and 100 U/ml penicillin/streptomycin (Sigma, USA) at 37 °C in 5% CO_2_ and 88% humidity. Cells viability was assessed by the trypan blue exclusion test.

To induce myogenic differentiation, cells were seeded at a concentration of 150,000 cells/well in 6-well plastic plates. After 48 h, cells at 60–80% confluence were washed once with serum-free DMEM, and the media were shifted to differentiation medium (DMEM supplemented with 2% heat-inactivated horse serum*)*. The media were changed every 24 h for 2, 3, or 6 days (Table 1).

**Table 1:** Mean values of FNDC5 gene expression fold change in pilot study after 3- and 12-h incubation

Groups	3 h incubation		12 h incubation	
	Value^a^	*P* value^b^	Value^a^	*P* value^b^
ATRA (0.1 μM)	0.50 ± 0.52	> 0.9	4.79 ± 4.86	0.9
ATRA (1.0 μM)	0.92 ± 0.45	> 0.9	16.09 ± 6.01	0.9
ATRA (10 μM)	3.90 ± 2.07	0.6	15.24 ± 21.39	0.4
Res (0.1 μM)	2.72 ± 2.16	0.9	15.68 ± 4.41	0.4
Res (1.0 μM)	4.15 ± 2.27	0.5	8.77 ± 5.60	0.4
Res (25 μM)	1.33 ± 0.82	> 0.9	11.90 ± 6.30	0.9
Res (50 μM)	4.15 ± 4.35	0.5	4.80 ± 3.87	0.7
Vehicle	1.05 ± 0.45	–	1.03 ± 0.32	–

^a^Data were represented as mean ± SD (*n* = 2)

^b^ Comparing each group with the vehicle group (The mean difference is significant at the 0.05 level)

Doses and incubation time in this study were obtained based on a pilot test. First, the effect of Res at concentrations (50, 25, 1 and 0.1 μM) and ATRA at concentrations (10, 1 and 0.1 μM) at 3 and 12 h was investigated on gene expression of Fndc5 (Table 2).

**Table 2:** Groups of main study using selected concentrations of Res and ATRA

Group 1	Res (25 μM)
Group 2	Res (1 μM)
Group 3	ATRA (10 μM)
Group 4	Res (12.5 μM) + ATRA (5 μM)
Group 5	Res (0.5 μM) + ATRA (5 μM)
Group 6	Res (25 μM) + ATRA (10 μM)
Group 7	Res (1 μM) + ATRA (10 μM)
Group 8	Vehicle

### RNA extraction, cDNA synthesis, and real-time polymerase chain reaction

After treatments, total RNA was extracted from C2C12 cells using RNeasy Plus mini kit (Qiagen, Valencia, CA) according to the manufacturer’s protocol. RNA samples were dissolved in DEPC-treated water. Their concentrations and qualifications determined by A260/A280 measurements using a spectrophotometer (NanoDrop Technologies, USA) and the values ranging from 1.9 to 2.1 were considered as acceptable. Synthesis of cDNA was performed using cDNA synthesis kit (Takara, Otsu, Japan) and used to FNDC5 mRNA expression by Real-time PCR (Applied Biosystem, Foster City, CA, USA) method. Real-time PCR was performed with SYBR Green (TaKaRa Ex Taq®) intercalating dye. Reactions were run in 40 cycles and two-step protocol, melting: 15 s at 95 °C, annealing, and extension: 60 s at 60 °C. After completion of amplification cycles, the melt curve was generated to verify if a single gene product had been amplified. Primer pairs for target genes were designed using Primer Express 3 software (Applied Biosystems, Foster City, CA, USA) and purchased from Metabion. Primer sequences are presented in Table 3. Normalizations of the target gene expression were done using the glyceraldehyde 3-phosphate dehydrogenase (GAPDH) gene expression level.

**Table 3:** Sequencing and information about primers

Gene Name	Sequence	Length	Tm	GC%
FNDC5(F)	ATGAAGGAGATGGGGAGGAA	20	54.50	50.00
FNDC5(R)	GCGGCAGAAGAGAGCTATAACA	22	55.30	50.00
GAPDH(F)	CACTGCCACCCAGAAGACTG	20	55.20	60.00
GAPDH(R)	CCAGTGAGCTTCCCGTTCAG	20	56.80	60.00

### Statistical analysis

Results represented at two independent experiments performed in duplicate. The calculation of the gene expression was based on the Paffl’s method. Data represented with the analysis of variance (ANOVA) mean and standard error for numerical variables. The mean gene expression was compared with test followed by Dunn’s post-hoc test. All statistical tests were two-sided and *P* < 0.05 was considered to be statistically significant.

## Results

### Gene expression of FNDC5 in pilot study after 3 h treatment by ATRA or res

**Fig. 1:**
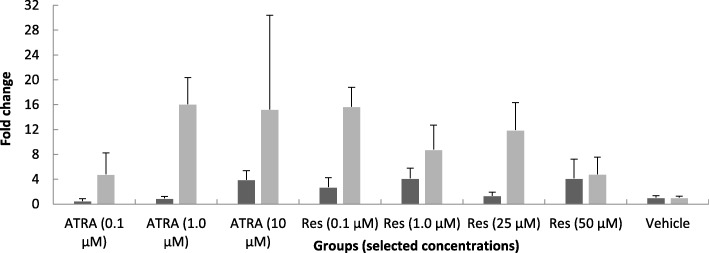
The effects of different concentrations of ATRA and Res on the fold change of FNDC5 gene expression in C2C12 cells after 3 and 12 h incubation in the pilot study

The expression of FNDC5 in C2C12 cells was evaluated. The real-time PCR analysis demonstrated that after 3 h treatment, neither ATRA nor Res could induce mRNA expression of FNDC5 gene. The difference between the effect of ATRA and Res treatments was not statistically significant (Table 1 and Fig. 1).

### Gene expression of FNDC5 in pilot study after 12 h treatment by ATRA or res

There was no significant increase in levels of FNDC5 expression with ATRA or Res at 12 h in pilot study. FNDC5 expression was not changed in all treatment groups compared to control. (Table 1 and Fig. 1).

### Combination treatments of ATRA and res

**Fig. 2:**
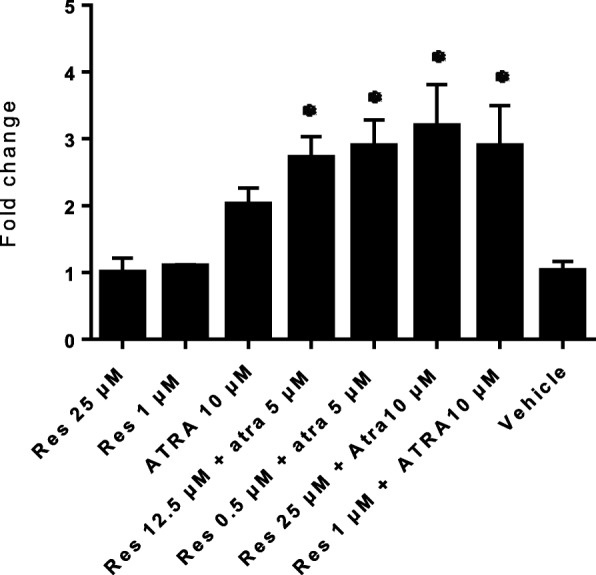
The effects of single and combined treatment of ATRA and Res in different concentrations on the fold change of FNDC5 gene expression in C2C12 cells after 12 h incubation

The effect of ATRA and its combination with Res and the synergistic and additive effects on FNDC5 expression in C2C12 Cells were explored. When the C2C12 was treated with half doses of Res and ATRA the expression of FNDC5 gene significantly elevated. The graph of each compound alone and in combination is shown in Fig. 1. These results are indicative of strong synergistic and additive effects with ATRA and Res (Tables 4, 5 and 6, Fig. 2).

**Table 4:** Mean and standard error of FNDC5 gene expression fold change in the main study (After 12 h incubation)

Groups	Mean	S.E	P value^b^
Res (25 μM)	1.01	0.21	*P* > 9.0
Res (1 μM)	1.1	0.01	P > 9.0
ATRA (10 μM)	2.02	0.24	0.299
Res (12.5 μM) + ATRA (5 μM)	2.72	0.31	^a^0.021
Res (0.5 μM) + ATRA (5 μM)	2.90	0.39	^a^0.009
Res (25 μM) + ATRA (10 μM)	3.20	0.61	^a^0.002
Res (1 μM) + ATRA (10 μM)	2.90	0.60	^a^0.010
Vehicle	1.03	0.13	–

^a^The mean difference is significant at the 0.05 levels

^b^ Comparing each group with the vehicle group.

**Table 5:** Pairwise comparison the effect of single and half doses of Res and ATRA on FNDC5 expression in C2C12 by Turkey analysis

		Mean Difference	95% Confidence Interval		Sig.
			Lower Bound	Upper Bound	
Res 25	Res (12.5 μM) + ATRA (5 μM)	−1.64^a^	−2.74	−.53	.003^a^
	Res (0.5 μM) + ATRA (5 μM)	− 1.79^a^	− 2.90	−.68	.001^a^
Res 1	Res (12.5 μM) + ATRA (5 μM)	− 1.52^a^	−2.62	−.41	.005^a^
	Res (0.5 μM) + ATRA (5 μM)	− 1.67^a^	− 2.78	−.56	.002^a^
ATRA 10	Res (12.5 μM) + ATRA (5 μM)	−.63	− 1.74	.47	.426
	Res (0.5 μM) + ATRA (5 μM)	−.78	− 1.89	.31	.232

^a^ The mean difference is significant at the 0.05 level

**Table 6:** Pairwise comparison the effect of single and combined doses of Res and ATRA on FNDC5 expression in C2C12 by Turkey analysis

		Mean Difference	95% Confidence Interval		Sig.
			Lower Bound	Upper Bound	
Res 25	Res (25 μM) + ATRA (10 μM)	−2.08^a^	−3.8162	−.3620	.015^a^
	Res (1 μM) + ATRA (10 μM)	− 1.77^a^	− 3.5007	−.0465	.043^a^
Res 1	Res (25 μM) + ATRA (10 μM)	− 1.96^a^	−3.6953	−.2411	.022^a^
	Res (1 μM) + ATRA (10 μM)	− 1.65	− 3.3798	.0743	.064
ATRA 10	Res (25 μM) + ATRA (10 μM)	− 1.08	− 2.8079	.6462	.343
	Res (1 μM) + ATRA (10 μM)	−.76	− 2.4924	.9617	.655

^a^ The mean difference is significant at the 0.05 level

Comparison of the results of single therapy with combination treatments, showed that in combination treatment with the half dose of each nutrient in the single therapy, had the best effect that indicating the synergistic effects of these two compounds (Tables 5 and 6).

## Discussion

The results of this study showed that combined treatments of ATRA and Res increased the expression of FNDC5 in C2C12 cells compared to control groups. To our knowledge, the present study is the first to demonstrate the ability of co-treatment of Res and ATRA in upregulation of FNDC5 gene expression. The gene expression of FNDC5 in single treatments of various concentrations of ATRA and Res was also upregulated but these increases were not statistically significant.

One of the advantages of combination therapy is that fewer doses of each drug can be used, which can help to reduce the possible side effects of medications.

Res known as a natural polyphenol that has anti-obesity effect due to activation of UCP-1 [33]. There is evidence that shows Res in 1–10 μM can inhibit differentiation of adipogenic cells into preadipocytes [34]. Andrade, et al. observed that oral administration of Res in mouse promoted thermogenesis in adipose tissue by increasing UCP-1 [31]. Retinoic acid (RA) also has anti-obesity effect by counteracting adipogenesis and promoting energy expenditure. Several studies highlighted this role for RA and several mechanisms have been proposed [35–37] . Stimulation of adrenergic pathway is a main trigger for thermogenesis [38]. In a recent study ATRA can upregulate FNDC5 expression at the same dose level of our study (10 μM) but in the longer duration (24 h) [18]. Res is an adrenergic receptor agonist that increases intracellular cyclic adenosine monophosphate (cAMP) levels [26]. Furthermore Res enhances cAMP by inhibiting phosphodiesterase4 (PDE4) [22]. cAMP increases the protein expression level of PGC-1α by two ways. First is sirtuin 1 (SIRT1) that affect PGC-1α activity through phosphorylation and acetylation [23, 24]. The second way is related to protein kinase A (PKA) and cAMP response element binding protein (CREB). The cAMP signalling pathway activates CREB through PKA. Then, the transcription factor CREB activates the expression of PGC-1α gene [21]. Therefore, Res is able to upregulate the expression of PGC-1α [39, 40]. It has reported that ATRA can also increase the PGC-1α expression through two pathways. First is Mitogen-activated protein kinase (MAPK)- Extracellular Signal-regulated Kinase-1 (ERK) - CREB and second is MAPK-p38-CREB activation [41]. An increase in expression of PGC-1α can enhance the expression of FNDC5 [42]. In accordance with these evidences, the results of the present study for the first time showed that Res and ATRA in additive and synergistic manner could increase the expression of FNDC5 (irisin) gene.

Since the discovery of irisin which is able to induction of thermogenesis, a few studies investigated the correlation of this myokine and nutrients. The obtained results in previous studies showed undesirable fatty acid profile is leading to high plasma irisin in children, especially in obese children. This study suggested the role of irisin in protection against metabolic disturbance [43]. In a previous study, the effect of vitamin A on FNDC5 gene expression has been investigated and the results showed that vitamin A can increase gene expression of FNDC5 through the ERK1/2-CREB-PGC1α-FNDC5 pathway [44]. Another study observed no association between 28 days vitamin D supplementation in a single dose (100.000 IU) and level of plasma irisin. Indeed, vitamin D could not effect on plasma irisin in human adults [45]. Seo et al. found no effect of treatment with aged garlic extract on the production of irisin in mouse skeletal muscle cells in vitro [46].

The results of the present study were consistent with previous reports that showed vitamin A promoted irisin production. In spite of these findings, additional studies are necessary to determine if ATRA and Res would be as effective as the in vitro model presented here.

Empirical studies are usually performed in at least three independent expriments in triplicate tests. In the present study, two independent experiments were performed and all experiments were carried out in duplicate due to budget constraints. This limitation may explain the high variation of the data and achievement of a non-significant increase in FNDC5 gene expression in some experiment.

## Conclusion

The result of this study has further elucidated the effects of single and combined treatment of ATRA and Res on the FNDC5 expression and metabolism of skeletal muscle adipocyte. In this study, the benefit of combination therapy was also evaluated. Taken together the nutrients supplement with the effect of thermogenesis have the potential to make a positive effect on obesity and the results of this study may reveal novel compounds in this line of investigation.

## Data Availability

The datasets used and/or analyzed during the current study are available from the corresponding author on reasonable request.

## Abbreviations

ANOVA: Analysis of variance
ATRA: All-trans retinoic acid
BAT: Brite/beige adipocyte
cAMP: Cyclic adenosine monophosphate
cDNA: Complementary DNA
CREB: CAMP response element binding protein
ERK: Extracellular Signal-regulated Kinase-1
FNDC5: Fibronectin type III domain containing-5
MAPK: Mitogen-activated protein kinase
PDE4: Phosphodiesterase4
PGC-1α: Proliferator-activated receptor γ coactivator-1α
PKA: Protein kinase A
PPAR: Peroxisome proliferator-activated receptor
Res: Resveratrol
SIRT1: Sirtuin 1
UCP-1: Uncoupling protein-1
WAT: White adipocyte
